# Selections and Abstracts

**Published:** 1916-11

**Authors:** 


### SELECTIONS AND ABSTRACTS

### NOTES ON THE TREATMENT OF WOUNDED AT THE AMERICAN AMBULANCE NEUILLY, SUR SEINE, FRANCE.*

By S. P. Oast, M. D. ,Portsmouth, Va.

The American Ambulance at Neuilly, under the direction of Dr. Winchester, Du. Bouchet, of Paris, and Dr. Jas. B. Hutchinson, of Philadelphia, occupies a large, thoroughly modern brick building, which was in course of construction for the purposes of a high school at the outbreak of the war, and shortly afterward completed and turned into a surgical hospital with accommodation for six hundred patients. It is admirably suited to its present purposes, and leaves but little to be desired in the way of structural alterations to make it ideal for surgical base hospital work.

In the spring and summer months, just past, during which time I was one of the resident physicians there, the wounded were being received from the Verdun and Somme regions on hospital trains, the journey being about ten hours.

The conditions with which we were occupied for the most part consisted of compound fractures of the extremities, extensive mutilating wounds, with loss of tissue substance in every possible situation; perforating and penetrating wounds, and recent amputations. Fully ninety per cent. of these lesions were produced by fragments of high explosive shells, the remainder being due to hand grenades, rifle balls and shrapnel, of frequency in the order named.

The treatment of wounds in general is the first thing I wish to mention.

The lesions, at the time of being received at the Ambulance, had existed on an average, I should say, of four or five days, this time being passed in the dressing stations and various temperary hospitals in the army zone. Most of the dressings were two and three days old, often older, and the wounds, nearly in every instance, were extensively infected, sloughing, and frankly purulent. The chief causes of infection were, for the most part, the ordinary pyogenic organisms (the streptococci, staphylococci, and proteus bacillus), but occasionally the gas producing bacillus, perfringens, was found to be the cause. Previously, during the Battle of Champagne, in the fall of 1915, and again on one or two other occasions, but to a less degree, there had been epidemics of wound infection by the gas bacillus, but at the time of my stay there were only a few cases, and these not severe. I know of but one amputation because of so-called "gas gangrene," occurring during my entire service of four and one-half months.

The usual plan pursued with all cases, no matter how trivial in external appearances, was to take the patient to the operating room within a few hours after admission, give ether, clean the wound thoroughly, remove as far as practicable all sloughy material and foreign bodies and, after making ample counter-openings for dependent through-and-through drainage, tubes were inserted for this purpose. In most cases, several small fenestrated tubes, soft and about the size of a number fifteen French catheter, as advised by Dr. Carrel, were used in place of the single large tube ordinarily used for drainage purposes. The ends of these small tubes were left protruding from the wound for four or five inches, care being taken that none of the fenestrations were without the wounds. Every two hours irrigations were done through these tubes, one end being closed in order to direct the fluid through the fenestrations, thus distending the wound cavity and forcing the fluid into the recesses of the tissues.

The dilute solution of sodium hypochlorite, or ordinary bleaching powder, prepared according to the formula of Dr. H. D. Dakin, of New York, was used routinely, until suppuration had subsided; then normal salt solution was substituted, and the wound allowed to fill in by granulation.

It will be seen that this is not exactly the method of wound treatment as practiced by Dr. Carrel, at Compiegne, though partaking somewhat of it. Everyone who has visited the Carrel hospital, and observed the results obtained by the treatment there, is very enthusiastic about it. It is there that closure of wounds by suture is practiced a good deal, after two or three days' treatment, with marked success. It has been found impracticable to follow this procedure at Neuilly, and the difficulty seems to lie in the longer duration of the lesions when received there.

Dr. Carrel's hospital occupies a very peculiar position, in that it possesses the facilities and equipment of a base hospital, at the same time being right on the front, within a few miles of the fighting. Here, the wounded are brought in, and treatment instituted within a few hours of the time of injury, before the infection has taken hold, while further back at Paris, the wounds are three, four and five days old when received, and stubbornly infected.

The method of irrigating wounds under slight pressure with Dakin's fluid, as set forth above, was best carried out in such conditions as compound fractures of the long bones, with through and through drainage. However, experience seemed to show that in areas in which the infection had become well established, as was the rule with our cases, this treatment seemed to possess no particular advantage over the older method of simple irrigation either with Dakin's fluid or other substance.

In conditions received late, the most appreciable effects of Dakin's fluid were seen in large open flesh wounds with extensive sloughing. These were cleaned up in a remarkably short space of time by the use of this solution, either as a Murphy drip or by fourth hourly dressings. Some caution must be exercised in its use, however, in the vicinity of large vessels, as an increasing number of severe secondary hemorrhages have occurred since its introduction and suspicion fastens very strongly to it as a factor in their production. Though the advocates of this solution claim that it is non-irritating, experience at the Ambulance has shown that any continued contact with the skin over a few days sets up a dermatitis, which has proved quite distressing in several instances. To prevent this, a protective layer of vaseline gauze was used next to the skin with complete satisfaction.

On the whole, Dakin's solution, chiefly because of its inexpensiveness, and also because of its efficacy, proved the most useful substance for general wound dressing, but it was thought that the frequent changing of dressings played an equal, or even greater, part in quickly cleaning up a wound, than any remarkable virtue of the fluid itself.

In the treatment of compound fractures, which forms the bulk of the work at Neuilly, many forms of apparatus were in use, the most noteworthy feature of which was the employment of the overhead suspension principle, by means of a wooden framework attached to the ends of the bed. This, as we know, is by no means new with the war, but its advantages were never so well appreciated until Dr. Blake began using it with his metal rod splint for fractures of the femur during the early days of the war in 1914. Since that time, it has been steadily gaining in favor, not only for fractures of the femur, but for fractures of the leg, arm, and forearm as fell.

I shall not attempt any lengthy description of it here, as it is doubtless familiar to all· Briefly, it serves these purposes: (1) To increase greatly the comfort of the patient in allowing a greater range of freedom in bed; (2) to decrease edema; (3) to facilitate dressings and the care of the patient generally.

It is not to be understood that overhead suspension introduces any system that replaces or interferes with extension and abduction, but allows and facilitates the employment of these principles, as before, by adhesive plaster strips, hitches, or Steinman's pins, with weights on the foot of the bed.

On the special form of apparatus in use at the Ambulance, none seems to deserve mention more than the ambulatory extension splint for the treatment of compound fractures of the humerus, devised by Dr. Levya, one of the residents at the Ambulance. This is an iron frame splint riveted to a plaster of Paris or aluminum plate jacket which encircles the chest and abdomen and, being adjustable, allows for any desired position of the arms and forearm, at the same time permitting extension on the lower fragment by means of a strong rubber band.

The results obtained by the use of this apparatus were very satisfactory, and seemed in every way to justify its use after the acuteness of the infection had subsided, in preference to keeping the arm suspended with the patient in bed.

This splint is an elaboration of what is known as the aeroplant splint, a device which has been in use at the Ambulance for some time, in the ambulatory treatment of various conditions about the shoulder, not calling for extension.

Compound fractures of the femur which, from their frequency of occurrence and gravity, deserve special consideration, were treated in a slightly modified form of the Blake splint, which consisted of two wrought iron rods, slightly longer than the leg, and connected at the lower end by a wooden cross piece, and at the upper end and middle by flat metal bands made to curve over the leg. The leg was cradled between these two iron rods by means of canton flannel straps, or, as in the region next the wound, by gutta percha tissue, and the whole suspended by tackle from the trestle work overhead. Extension was made on the lower fragment and leg by weights on the foot of the bed, attached by long broad straps applied to both sides of the legs and thigh as high up as the position of the wounds would permit. These straps were either of adhesive plaster or canton flannel, in which latter case they were applied with a special form of glue. In a few cases, because of the location of the wounds, or of irritation of the skin, extension was effected through the medium of a hitch around the semi-flexed knee, using a broad piece of muslin over a very thick pad of cotton. It was found quite essential to have this pad of cotton very capious to prevent cutting the skin.

Dr. Hutchinson was not very partial to the use of Steinman's pins in compound fractures of the femur because of trouble with infection, and they were not being used, but I remember one case of simple fracture in which a steel pin, resembling a meat skewer (no Steinman pins being available), was driven through the femur just above the condyles, and extension made by this means with an excellent result. As to the amount of weight used, it was the usual plan to run the total up to twenty pounds in the course of the first few days; then, after three or four weeks, after the shortening had been overcome, to reduce this amount by a few pounds.

Abduction was obtained by means of a horizontal wooden bar nailed on the uprights at the foot of the bed, the degree being determined largely by the level of the fracture, but the leaning was to abduct well in every case.

It will not cause much surprise when I say that, of all the conditions met with at Neuilly, by far the most troublesome were compound fractures of the femur. The chief difficulty was the inability to control the infection; to prevent pocketing in the abundant fleshy tissues of the thigh. Wide through and through drainage was established in these cases immediately after arrival, and irrgations and dresings practiced very frequently, but no plan of treatment could be found to prevent the burrowing, and it was not unusual for the case to make three and four trips to the operating room, before the situation could be gotten in hand, and not always then.

The mortality rate in surgical base hospitals has been surprisingly low during the war, estimated at something over two per cent., I believe, and if the Ambulance at Neuilly can be taken as an index, I think it is safe to say that fully one-fourth of these deaths are due to compound fractures of the femur with their attendant complications of infection and secondary hemorrhage.

One meets with such a great variety of conditions in a war hospital of the character of the Ambulance at Neuilly, that it is obviously out of the question to attempt anything but a very hasty review of some of the more important ones here.

Among other things, I should like to mention compound fractures near or involving the ankle joint. A form of apparatus known as a crade was generally used for the treatment of these conditions, though some times an old-fashioned fracture-box seemed to meet the requirements better than anything else. The cradle, mentioned above, was an appliance, by means of which the leg and foot were slung in a canvas hammock between two parallel wooden bars, and made to slide on a framework of wood by the use of a trolley arrangement. This permitted of extension, when practicable, and possessed the advantages over a fracture-box of allowing greater freedom of movement and comfort to the patient, and at the same time permitting the dressing to be made with little or no disturbance to the position of the fragments.

The metohd of treating recent amputations was very interesting. Because of conditions which obtained on the front, the French army surgeons had adopted, as most practicable, the procedure of amputating without flaps—"chop amputations," as they are frequently called. This, of course, was a reversion to old antiseptic methods, and these cases when they arrived at the Ambulance were invariably badly infected.

The plan pursued here was to clean up the wound by using Dakin's solution, either as a drip or by frequent dressings. This took on an average of three or four days, after which four adhesive plaster straps were applied to the skin in such a way as to encircle the circumference and, with the aid of a spreader, equal traction was made on all four of these straps by weights on the foot of the bed, as in Buck's extension. In this way flaps were produced, which were drawn well down below the end of the bone. The procedure then was to apply adhesive plaster fitted with eyelets to these flap margins, and further reduce the granulating area by firm lacing. It was by such measures that a great saving of time was brought about in these otherwise very tedious cases, usually about three weeks sufficing to see the stump entirely closed over.

This method of lacing wound edges together also found general application to large granulating areas in almost any situation. In a number of cases, marked for skin grafting, it was found that such an operation was unnecessary after the vigorous employment of lacing.

Before I stop, I should like to mention the nerve suturing work of Dr. Hutchinson, which was showing some very promising restorations of functions, even at the early stage of my stay. Also, I should like to call attention to the successful character of the plastic work on the face being done by Drs. Hart and Powers in collaboration with the dental department, under the direction of Dr. Hays and Davenport of Paris.—The Virginia Medical Semi-Monthly, December 8, 1916.

### A SERUM DIAGNOSIS OF ACTIVE TUBERCULOSIS.

It is a general clinical experience that there are several groups of cases in which it is extremely difficult to establish or to exclude an active tuberculosis. Such cases are certain types of chronic bone and joint disease; the adenopathies that may arise from blood dyscracias, neoplasm, Hodkin's disease, syphilis or tuberculosis; peritoneal infections of unknown etiology; thoracic affections that may mean syphilis, tuberculosis or new growth; renal affections in which tuberculosis is strongly suspected but may not be demonstrable; obscure acute fevers in which miliary tuberculosis is always to be borne in mind but is notoriously dfficult to establish ante-mortem; cases of suspected incipient pulmonary tuberculosis,etc. All these conditions point the need for some clinical or laboratory procedure that will definitely determine the presence or absence of an active tuberculosis.

Many tests have been proposed for the early diagnosis of tuberculosis,—the urochromogen test, the Arneth leucocyte count, the employment of tub.erculin in various ways, etc. In spite of a rather voluminous literature concerning these methods, the outstanding fact remained that clinicians could find but comparatively small usefulness for them·

Following upon the establishment of Wassermann's epoch-making serum reaction test for syphilis, many attempts were made to establish for tuberculosis a similar complement-fixation test. In fact, Wassermann himself, with his co-workers, tried such a reaction ,adapted for tuberculosis, and soon thereafter many investigators described similar tests with suggestive results. Except for intermittent studies during the past decade, this subject assumed no especial importance, however, until very recent years, when it was quickened to new interest by the published work of Calmette, Radcliffe and others, Besredka and his followers (Inman, Debains and Jupille, Renaux, Bronfenbrenner), Craig and Petroff. These workers showed that complement-fixation in tuberculosis could give a high percentage of positive reactions in positive cases but not infrequently the reacton was also positive in "healed" or "arrested" cases, in syphilitics who had no evidence of tuberculosis, and in some normal individuals.

This year Hymen Hiller and Hans Zinsser announced in the Proceedings of the Society for Experimental Biology and Medicine, July, 1916, the successful application of a method for the serum diagnosis of tuberculosis based upon complement-fixation in the presence of a dry salt extraction of tubercle bacilli used as an antigen. The results with this preparation as published by Miller (Journal of the American Medical Association, November 19, 1916), have been very striking. A high percentage of positive reactions is obtained in positive cases. Only about five per cent. of cases clinically declared "arrested" failed to give negative reactions. (In such cases the von Pirquet test, for example, gives about 100 per cent. positive reactions). There is no cross-fixation with the serums of luetic or normal people.

A positive reaction with Miller's test indicates absorption from some active focus. Unlike the tuberculin tests it does not denote the mere presence of a tuberculoss focus, healed or unhealed. A positive reaction is, therefore, an indication for treatment, surgical or medical. What is also very important, Miller has shown that as the patient approaches recovery the reaction loses its intensity, disappearing when the disease is cured.

Miller's test is still young and awaits further trial. It has, however, been employed critically in various institutions, where it has been found quite as useful and reliable as the Wassermann test for syphilis.

This appears then to be another fine accomplishment of the research laboratory, one quite as welcome to the surgeon as to the internist in demonstrating the presence of an active tuberculosis, in aiding prognoses, in determining whether a lesion has been cured.—Editorial American Journal of Surgery.

### RESUSCIATION IN VARIOUS FORMS OF ASPHYXIA.

It not rarely falls to the lot of a medical man to be called upon to carry out artificial respiration, because of a complication arising from a surgical operation, in cases of drowning, or when illuminating or coal gas has been inhaled. Many years ago the writer of this article and Dr. Martin carried out a research upon the quantity of air which could be propelled into and out of the lungs by Marshall-Hall and Sylvester methods, and found that the latter ways more efficient than the Marshall-Hall procedure. Since then Schafer has brought forward his method of artificial respiration, in which, it will be recalled, the patient lies with the anterior surface of the body downward and the head turned to one side. The operator then kneels astride of the patient's loins and by pressure upon the floating ribs, just above the kidneys, cause ingress and egress of air from the chest. Schafer seems to have proved that this method moves a greater volume of air than the two older ones, and he and others who have investigated it in England have heartily recommended it for the resuscitation of the apparently drowned. In our own limited experience with it, it has seemed disadvantageous in that it is more prone than the other methods to dislodge the contents of the stomach, pumping them into the esophagus and pharynx, and so causes their introduction into the air-passages. On the other hand, it can well be urged that the posture of the patient and the movements of the physician can more readily displace fluid from the bronchial tubes when this method is followed than when the patient lies upon the back as in the Marshall-Hall or Sylvester methods.

Many years ago Fell introduced a mechanical apparatus for the development of artificial respiration which, in some respects, resembled those forms of apparatus which are used in the physiological laboratories for the maintenance of artificial respiration in animals, and since that time a number of more or less complicated mechanical devices, to which such names as pulmotor and lungmotor has been applied, have been invented. In the use of all of these an endeavor is made to provide the patient not only with air but with oxygen as well, and most of them have gauges and valves whereby the supply of air and oxygen can be readily adjusted. In cases of apparent drowning such apparatus cannot be as useful as the older forms of artificial respiration, since it does not tend to pump the liquid out of the lung, but, on the contrary, may serve to drive it into the smaller bronchioles, where it will do damage and from which it cannot be displaced. Furthermore, if these mechanical devices are not used by those who are skilful in their employment there is a chance that too high pressure will be employed and that artificial emphysema will ensue.

In this connection an investigation which has been carried out by Henderson is of considerable importance and interest. He points out that superintendents of hospitals, mines, gas works, electric light and telephone companies, fire departments, and persons in charge of swimming places are continually asking for advice as to the relative merits of the different kinds of apparatus which are now on the market, which are variously called pulmotors, lungmotors, etc. As a result of a consideration of all the forms of apparatus which are commonly employed he concludes that approved, prompt manual methods of artificial respiration will accomplish more for resuscitation from drowning, electric shock, or asphyxia than is possible with any form of apparatus. One of the reasons for this probably lies in the fact that the application of the hands to the chest induces what has been called in Europe "cardiac massage," or, in other words, exercises an effect upon the congested right heart and the engorged great veins of the chest. It is also important that those who are called upon to resuscitate persons suffering from asphyxia shall be thoroughly trained in the use of the manual methods, since the delay in sending for mechanical devices may be fatal. In other words, every minute that passes after respiration has apparently ceased greatly diminishes the chance for resuscitation, and while a proper apparatus correctly used and immediately employed in suitable cases may be better than the manual method, practical experience shows that it is rarely immediately at hand.

In gas and smoke cases Dr. Martin and the writer proved, many years ago, that the addition of oxygen was mose efficacious, and if a complete artificial respiration apparatus cannot be had oxygen gas should be freely given to the patient, mixed with atmospheric air, care being taken, however, that it is not driven into the lungs under such pressure as to induce distention and emphysema.—The Therapeutic Gazette.

